# The Effect of Shelter-in-Place Orders on Social Distancing and the Spread of the COVID-19 Pandemic: A Study of Texas

**DOI:** 10.3389/fpubh.2020.596607

**Published:** 2020-11-26

**Authors:** Marco A. Castaneda, Meryem Saygili

**Affiliations:** Department of Social Sciences, University of Texas at Tyler, Tyler, TX, United States

**Keywords:** COVID-19, shelter in place, social distancing, public health, pandemic (COVID-19)

## Abstract

**Objectives:** We study how the state-wide shelter-in-place order affected social distancing and the number of cases and deaths in Texas.

**Methods:** We use daily data at the county level. The COVID-19 cases and fatalities data are from the New York Times. Social distancing measures are from SafeGraph. Both data are retrieved from the Unfolded Studio website. The county-level COVID-related policy responses are from the National Association of Counties. We use an event-study design and regression analysis to estimate the effect of the state-wide shelter-in-place order on social distancing and the number of cases and deaths.

**Results:** We find that the growth rate of cases and deaths is significantly lower during the policy period when the percentage of the population that stays at home is highest. The crucial question is whether the policy has a causal impact on the sheltering percentages. The fact that some counties in Texas adopted local restrictive policies well before the state-wide policy helps us address this question. We do not find evidence that this top-down restrictive policy increased the percentage of the population that exercised social distancing.

**Discussion:** Shelter-in-place policies are more effective at the local level and should go along with efforts to inform and update the public about the potential consequences of the disease and its current state in their localities.

## 1. Introduction

The global pandemic of COVID-19 affected countries and communities around the globe with detrimental impacts on population health as well as economies. The tragic examples from countries such as Italy and Spain showed the importance of flatting the curve as sudden surges in hospitalization can easily strain even the well-functioning healthcare systems (1, 2). COVID-19 is thought to spread mainly through close contact from person-to-person^1^. Therefore, policymakers tried to control the spread of the disease and reduce the burden on the healthcare system by policies that encourage and sometimes force social distancing^2^. In the United States, the state-wide shelter-in-place policies are the most common of such policies. Shelter-in-place orders (SIPO) require residents to remain home for all but essential activities such as purchasing food or medicine, caring for others, exercise, or traveling for employment deemed essential. Between March 19 and April 20, 2020, 40 states and the District of Columbia adopted SIPOs (3).

A fast-growing body of literature explores the potential impacts of stay-at-home orders on various indicators from the COVID-19 hospitalizations, cases, and fatalities to their implications for mental health (4, 5), physical health (6, 7), and domestic violence (8–10). Some studies find that stay-at-home orders are associated with a slower growth rate of hospitalization (11, 12). Closer to our study are the ones that focus on COVID cases and deaths. (13) analyzes the US data from March 1, 2020, to April 27, 2020, and find that adoption of government-imposed social distancing measures reduced the daily growth rate by 5.4 percentage points after 1–5 days, 6.8 after 6–10 days, 8.2 after 11–15 days, and 9.1 after 16–20 days. Similarly, (3) work with data from all the states from March 8, 2020, to April 17, 2020. Using daily state-level social distancing data from SafeGraph and a difference-in-differences approach, they find that adoption of a shelter-in-place order (SIPO) is associated with a 5–10% increase in the rate at which state residents remained in their homes full-time. Also, using daily state-level coronavirus case data collected by the CDC, they find that approximately 3 weeks following the adoption of a SIPO, cumulative COVID-19 cases fell by 44%. Similar to our paper, (14) focus on a particular state, namely California, to estimate the impact of the state-wide SIPO on the COVID-19 cases and deaths.

We focus on a large state, Texas. Our data span almost 4 months, which allows us to see better the evolution of the pandemic during as well as after the expiry of the SIPO. The fact that Texas is one of the relatively early states to start the reopening and experiences a post-opening surge in the number of cases makes it an interesting case to study closely. We use daily data from March 1, 2020 to June 27, 2020, to study the evolution of the COVID-19 pandemic in 254 Texas counties. We analyze the impact of the statewide shelter-in-place order issued on April 2, 2020, on the measures of social distancing, and consequently, on the COVID-19 cases and deaths.

## 2. Materials and Methods

### 2.1. Data

The data for this study comes from the Unfolded Studio website (https://covid19.unfolded.ai/). The website compiles data from various sources. The COVID-19 cases and fatalities data are from the New York Times^3^. Social distancing measures are from SafeGraph^4^. County-level population data are from the US Census. SafeGraph's social distancing data are generated using a panel of GPS pings from anonymous mobile devices. The data determine the common nighttime location of each device and call that location “home” (approximately a 153 by 153 m area). The shelter-in-place percentage reported in the Unfolded data shows the percentage of the population who stayed at home on a day. Finally, we use the National Association of Counties' (NACo) county explorer to get county-level COVID 19 related policy responses. Our dataset is daily and at the county level.

### 2.2. Methods

#### 2.2.1. Theoretical Background

We provide a basic description of the theoretical foundations for the spread of the disease. Although the model of a pandemic is not strictly exponential, the exponential growth model may provide a good approximation at the beginning of the pandemic and is implicitly used in much of the literature.

The discrete-time version of the exponential growth model is described by the equation^5^:

(1) $$c_{t}=c_{0}{\left(1+\delta \right)}^{t}$$

where *c*_*t*_ denotes the total number of cases at time *t*, δ represents the growth rate, and *c*_0_ is the number of cases at time zero. The parameter of interest is δ, the rate of growth of the process, which can be interpreted as the number of infected individuals by one infected individual in a day. Rearranging Equation (1), we can write

(2) $$\frac{c_{t+1}-c_{t}}{c_{t}}=\delta .$$

Given a sample {*c*_*t*_} of observations, we are interested in estimating the growth rate and the effect of a policy variable (*T*_*t*_) on the growth rate. This can be done by estimating the equation

(3) $$y_{t}=\beta_{1}+\alpha_{1}T_{t}+u_{t}$$

where

(4) $$y_{t}=\frac{c_{t+1}-c_{t}}{c_{t}}$$

where *y*_*t*_ is the growth rate, β_1_ is the estimated growth rate before the policy, and β_1_ + α_1_ represents the growth rate after the policy, with α_1_ being the effect of the policy.

The model we present provides a good approximation for the evolution of the number of cases. Fatalities, however, are much harder to model. Similar to cases, we analyze the growth rate of deaths to provide a complete picture. However, we are aware of the problems associated with fitting the same type of model for cases and fatalities. First, social distancing may have different effects on the growth of cases and deaths. In particular, social distancing measures are more likely to show a bigger and faster impact on the spread of the disease than the number (or growth rate) of fatalities. In addition, as medical researchers learn more about the disease, they will be able to reduce fatality rates, presumably, long before they find a definitive cure to completely eradicate it. Even though the disease keeps spreading, we may see lower rates of fatalities. This may even happen without any outside intervention if the virus evolves and becomes less fatal.

The analysis of the effects of the shelter-in-place order on social distancing and the number of cases and deaths was conducted at the state level and at the county level.

#### 2.2.2. State-Level Analysis

For the state-level analysis, we aggregated the county data by summing up the daily values for all counties to get the state-level time series data. To estimate the effects of the SIPO, we divide the SIPO period into 4 weekly subperiods since we expect the SIPO to have a gradual impact on the spread of disease^6^. We estimate the following empirical model:

(5) $$y_{t}=\beta_{0}+\alpha_{0}SIPO_{0}+\alpha_{1}SIPO_{1}+\alpha_{2}SIPO_{2}+\alpha_{3}SIPO_{3}+\theta_{1}Day+u_{t}$$

where *y*_*t*_ is either the growth rate of cases and fatalities, or the measure of social distancing. SIPO_0_ is the week the order was enacted, SIPO_1_ is the second, SIPO_2_ is the third week, and SIPO_3_ is the fourth and the last week that the policy was in effect. The model also includes a linear daily time trend. In addition, we estimate the effects of the SIPO on the growth rate of cases and fatalities controlling for social distancing to determine whether the policy had any effects other than its effect through social distancing.

#### 2.2.3. County-Level Analysis

The state-level analysis indicates that the SIPO reduced the spread of the disease through its effect on social distancing (the percentage of the population sheltering in place). Therefore, we use the county-level analysis to further investigate the effect of the statewide SIPO on social distancing, while controlling for local policies.

First, we investigate the effect of the statewide SIPO on the larger counties which implemented local policies before the statewide SIPO (Harris, Dallas, Tarrant, Bexar, or Travis). In each of these counties, first, a public health emergency is declared, which is then followed by a local stay-at-home order, and this is later followed by the state-wide shelter-in-place order. We assume each policy replaces the former. For the analysis, we estimated the following equation:

(6) $$\begin{array}{l} y_{i,t}=\beta_{0}+\alpha_{0}SIPO_{0}+\alpha_{1}SIPO_{1}+\alpha_{2}SIPO_{2}+\alpha_{3}SIPO_{3}+\beta_{1}X_{i,t}\\ \;+\theta_{1}Day+u_{it} \end{array}$$

where *y*_*i,t*_ is the sheltering percentages in county *i* on day *t* and *X*_*i,t*_ includes the county-level policies (local emergency or local shelter-in-place order). In addition, we control for a daily time trend and county fixed effects. The regressions are weighted by county-population and the standard errors are clustered at the county-level.

Next, we investigate the effect of the statewide SIPO on the counties that never had any type of local county-level policy. If the statewide SIPO is effective in increasing the percentage of the population that stays at home, one would expect counties without any restrictive local policies to catch up with other counties once the state-wide blanket policy is imposed. Therefore, we estimated the following equation including all the counties in the state:

(7) $$\begin{array}{l} y_{i,t}=\beta_{0}+\alpha_{0}SIPO_{0}+\alpha_{1}SIPO_{1}+\alpha_{2}SIPO_{2}+\alpha_{3}SIPO_{3}+\\ \alpha_{4}SIPO_{0}*NoPolicy+\alpha_{5}SIPO_{1}*NoPolicy+\alpha_{6}SIPO_{2}*NoPolicy+\\ \alpha_{7}SIPO_{3}*NoPolicy+Day_{t}+u_{i,t} \end{array}$$

where *y*_*i,t*_ is the sheltering percentages in county *i* on day *t*. The key variable, *NoPolicy*, is a dummy that takes the value of one if the county did not have any local policy prior to the statewide SIPO. We include day and county fixed-effects. The regressions are weighted by county population and the standard errors are clustered at the county-level.

## 3. Results

### 3.1. State-Level Results

**Figure 1:**
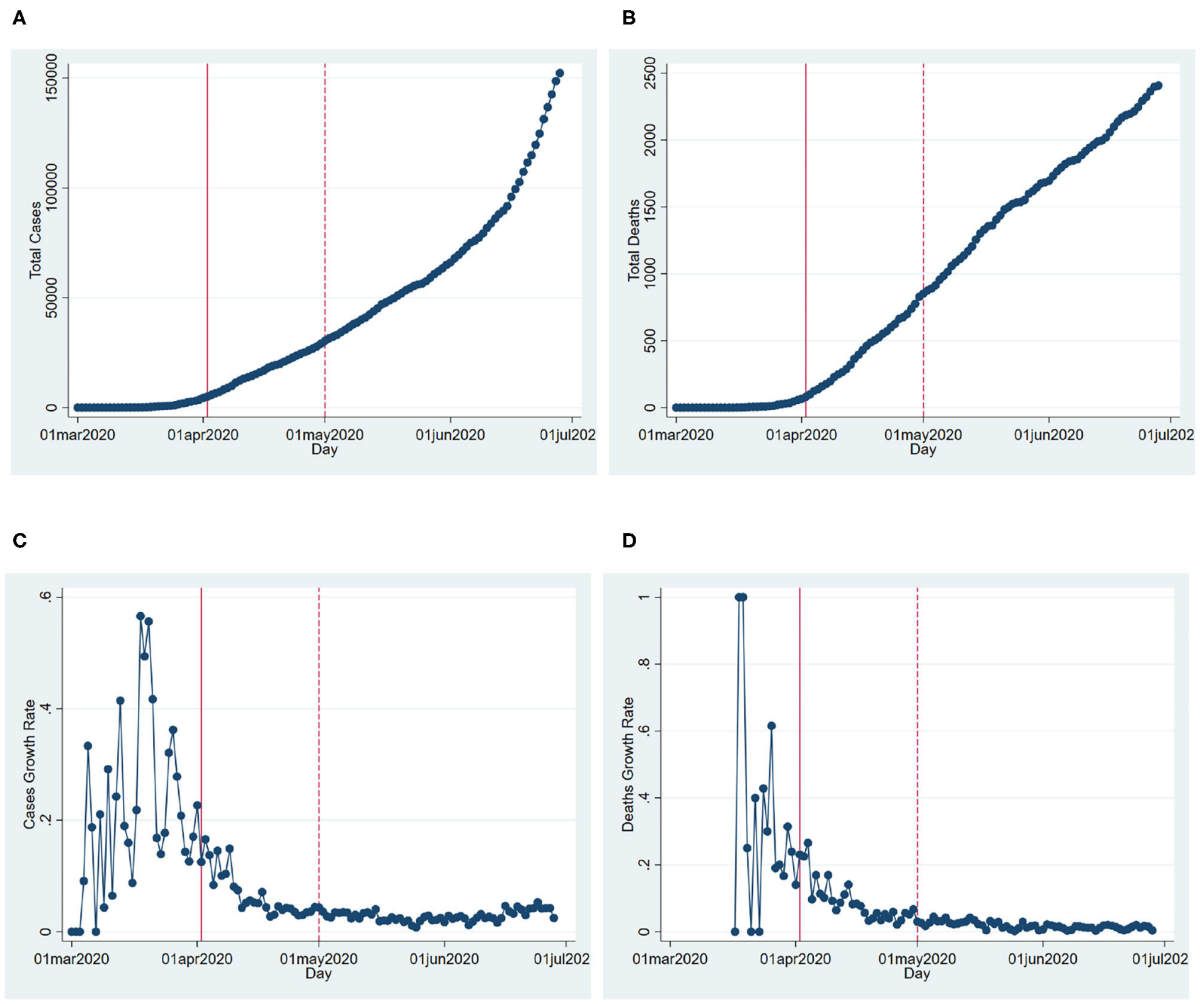
COVID-19 cases and fatalities in Texas. **(A)** Cumulative cases. **(B)** Cumulative fatalities. **(C)** Daily cases growth. **(D)** Daily fatalities growth. This figure shows the cumulative cases and fatalities, as well as their growth rates over the time in Texas. The solid and dashed vertical lines show the beginning (April 2, 2020) and the end of the state-wide shelter-in-place order (May 1, 2020).

Figure 1 shows the total cases and fatalities by day in Texas as well as the growth rates of those. We estimate Equation (5) for the growth rate of cases and fatalities. The results in Table 1 show that the growth rates of cases are 0.07–0.084 points lower during the SIPO period. Given that the mean growth rate of cases is about 0.1 outside the period, these numbers imply large drops in the growth rates of cases during the period. Similarly, the growth rate of deaths is 0.105–0.131 points lower during the SIPO, the largest decreases happening in the third week of the policy.

**Table 1:** The growth of COVID-19 cases and fatalities.

	**Cases**	**Fatalities**
SIPO_0_	−0.035	−0.067
	(0.023)	(0.061)
SIPO_1_	−0.070^***^	−0.105^**^
	(0.022)	(0.051)
SIPO_2_	−0.084^***^	−0.131^***^
	(0.016)	(0.043)
SIPO_3_	−0.074^***^	−0.108^***^
	(0.013)	(0.036)
Day	−0.002^***^	−0.004^***^
	(0.000)	(0.001)
Observations	117	102
Adjusted R^2^	0.390	0.368

***, ** *Indicate significance at 1% and 5%, respectively. Robust standard errors are reported in parentheses*.

**Figure 2:**
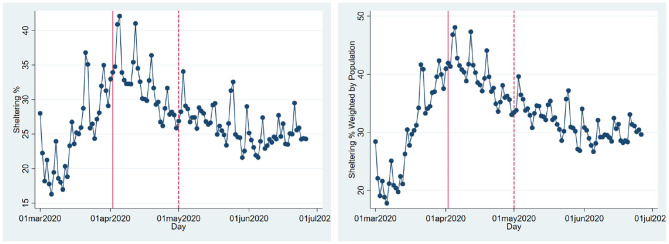
Sheltering population. **(A)** Sheltering %. **(B)** Sheltering % weighted by population. This figure shows the average percentage of the population that stays at home in Texas over time. The solid and dashed vertical lines show the beginning (April 2, 2020) and the end of the statewide shelter-in-place order (May 1, 2020). For the right panel, the counties' sheltering percentages are weighted by the counties' populations, and then aggregated at the state level.

We also analyze the impact of the policy on social distancing measures. In particular, we look at the percentage of population sheltering in place created by SafeGraph. Figure 2 shows the percentage of the population that shelters in place. Table 2 reports the regression results we get from estimating Equation (5) for the sheltering percentages^7^. Both the table and figure indicate the percentage of the population that shelters in place is, on average, higher during the SIPO period. The highest rate of sheltering corresponds to the first week of the SIPO, and the sheltering percentage gradually goes down even when the SIPO was still in effect. Compared to the sheltering percentage outside the policy period (25%), the share of sheltering population is about 9–41% higher during the SIPO period.

**Table 2:** Sheltering population and the SIPO.

	**Sheltering %**
SIPO_0_	10.443^***^
	(1.591)
SIPO_1_	8.584^***^
	(1.411)
SIPO_2_	5.870^***^
	(1.067)
SIPO_3_	2.287^***^
	(0.784)
Day	0.010
	(0.013)
Observations	118
Adjusted R^2^	0.400

***, ** *Indicate significance at 1% and 5%, respectively. Robust standard errors are reported in parentheses*.

Table 3 shows the impact of the SIPO on the growth of cases and fatalities when we control for lagged sheltering percentages. Because of the nature of the disease, social distancing measures show impact only after a while. We estimate Equation (5) using the 14-day lagged sheltering percentages in addition to the SIPO indicators and time trends^8^. The table reveals that the SIPO dummies are statistically insignificant once the sheltering population is controlled for. These results suggest the SIPO may slow down the spread of the disease through its effect on the percentage of people sheltering in place. The policy itself is not significant once we control for this percentage.

**Table 3:** The growth rates controlling for lagged sheltering percentages.

	**Cases**	**Fatalities**
SIPO_0_	0.017	−0.018
	(0.025)	(0.051)
SIPO_1_	−0.006	−0.043
	(0.020)	(0.033)
SIPO_2_	0.020	−0.025
	(0.027)	(0.026)
SIPO_3_	0.006	−0.027
	(0.021)	(0.021)
Lagged sheltering	−0.009^***^	−0.009^**^
	(0.002)	(0.004)
Day	−0.002^***^	−0.003^***^
	(0.000)	(0.001)
Observations	118	102
Adjusted R^2^	0.442	0.409

***,** *Indicate significance at 1% and 5%, respectively. Robust standard errors are reported in parentheses*.

The state-level analysis shows one thing is clear: Social distancing slows down the growth of cases^9^. The important question remaining is whether the state-wide SIPO had a causal impact on social distancing as measured by shelter-in-place percentages. We address this question in the next section.

### 3.2. County-Level Results

Figure 2 shows that the shelter-in-place percentages started to increase well before the state-level SIPO order. According to NACo, many counties adopted local policies such as public health emergency and safer-at-home declarations days or weeks before the state-wide policy. Overall, 70 counties in Texas have at least one type of policy, emergency or county-level shelter-in-place order, while 30 have both. The adoption of these policies is unlikely to be random. In particular, the size of the population and the number of cases are positively correlated with the likelihood of restrictive policies. This is particularly true for county-level stay-at-home orders. Several counties declared a health emergency early on when the cases were few. As the cases went up, the counties with higher numbers of cases adopted shelter-in-place orders.

We take a close look at the county-level trends in the most populated five counties: Harris, Dallas, Tarrant, Bexar, and Travis. These span the majority of the four biggest cities in Texas: Houston (Harris), Dallas (Dallas and Tarrant), San Antonio (Bexar), and Austin (Travis). These five counties make about 44% of the total Texas population, while the remaining 249 counties make up the rest. Perhaps not surprisingly, the earliest COVID-19 cases emerged in these five counties. And, all of these counties ordered a county-level stay-at-home policy before the state-wide SIPO. They also declared a county-wide emergency even before the sheltering policies^10^.

**Figure 3:**
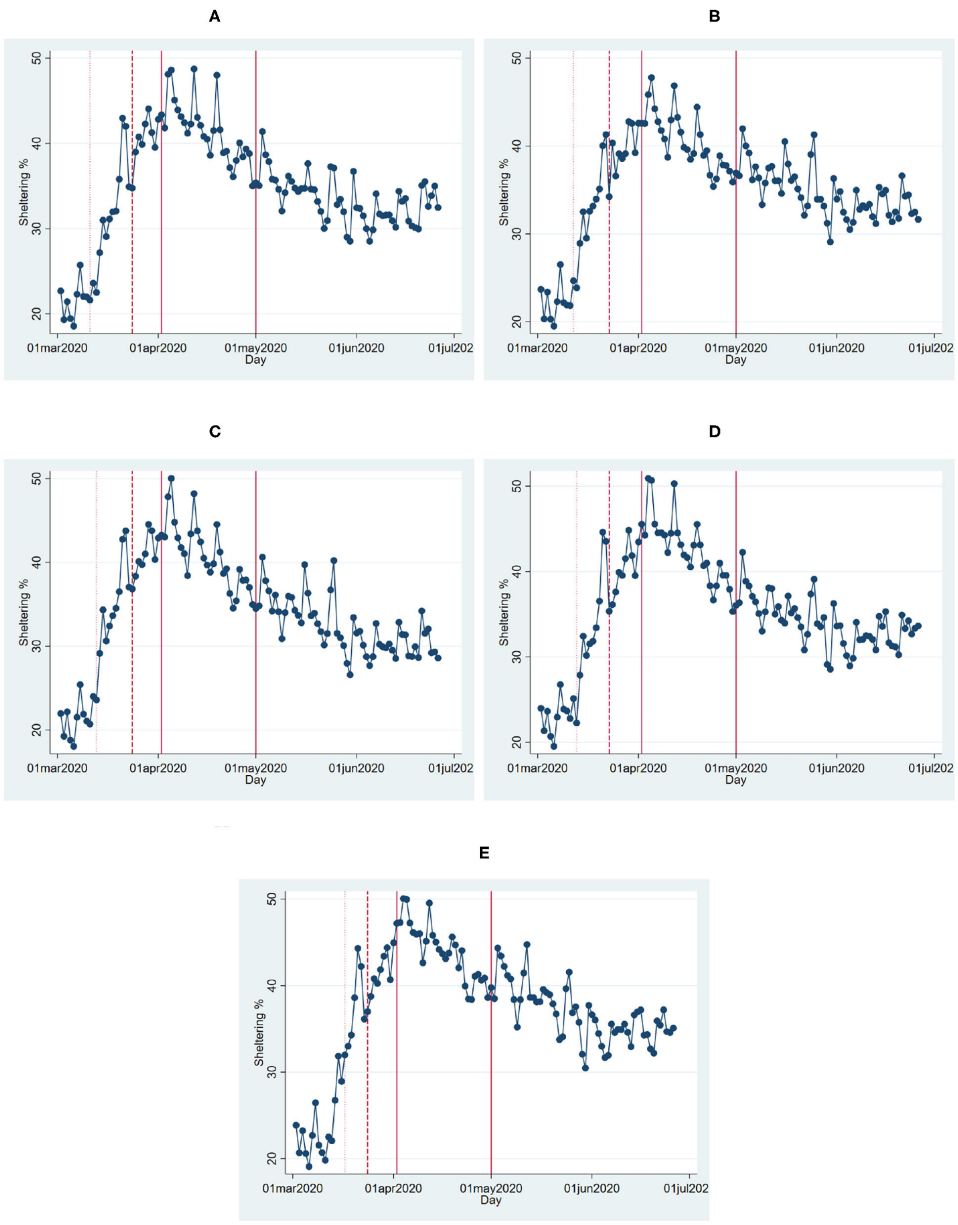
Sheltering percentages in Texas counties. **(A)** Harris. **(B)** Dallas. **(C)** Tarrant. **(D)** Bexar. **(E)** Travis. This figure shows the percentage of the population that stays at home in the five most populated counties of Texas over time. The dotted line shows the emergency declaration date, the dashed line shows the county-wide SIPO, the solid lines mark the beginning and the end of the statewide SIPO.

Figure 3 shows the percentage of the populations sheltering in place in each of these counties. Table 4 shows the results from estimating Equation (6). The results reveal that the initial pronounced increase in sheltering percentages corresponds to the declaration of county-wide emergency and shelter-in-place orders.

**Table 4:** Sheltering percentages in most-populated Texas counties.

	**Sheltering %**	**Sheltering %**	**Sheltering %**
Emergency			6.439^***^
			(0.765)
County SIPO		10.724^***^	12.998^***^
		(0.417)	(0.503)
SIPO_0_	13.122^***^	15.093^***^	17.095^***^
	(0.430)	(0.442)	(0.495)
SIPO_1_	10.839^***^	12.604^***^	14.373^***^
	(0.413)	(0.426)	(0.467)
SIPO_2_	8.690^***^	10.250^***^	11.787^***^
	(0.414)	(0.427)	(0.453)
SIPO_3_	4.938^***^	6.278^***^	7.566^***^
	(0.382)	(0.396)	(0.412)
Day	0.034^***^	0.063^***^	0.097^***^
	(0.006)	(0.006)	(0.004)
Observations	585	585	585
Adjusted R^2^	0.414	0.590	0.647

***, ** *Indicate significance at 1% and 5%, respectively. Robust standard errors are reported in parentheses*.

**Figure 4:**
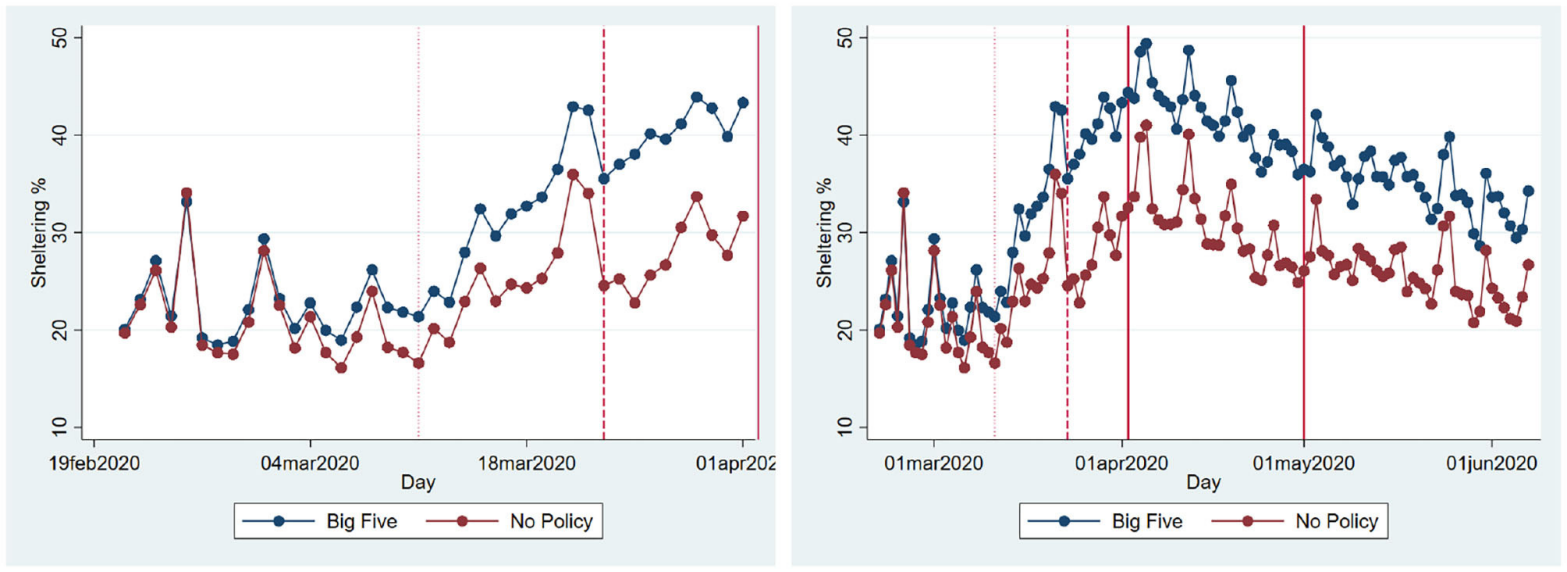
Sheltering percentages. **(A)** Before SIPO. **(B)** Whole period. The figure shows the average sheltering percentages in two groups of counties: The most populated five (Harris, Dallas, Tarrant, Bexar, and Travis) and the counties that never adopted any county-level policy. The dotted line shows the earliest emergency declaration date among the largest five (March 11, 2020), the dashed line shows the earliest county-wide SIPO (March 23, 2020), the solid lines mark the beginning and the end of the statewide SIPO.

Next, we take a look at the counties that never had any type of county-level policy. Figure 4 shows that there is a significant difference in terms of sheltering population in the biggest five counties vs. counties that never adopted any type of policy prior to the state-wide SIPO. The panel that shows the trends before the state SIPO reveals that in both groups of counties, the sheltering percentage increases over time. However, the gap widens over time. Interestingly, even after the implementation of the state SIPO, the gap persisted. The policy did not disproportionately affect the counties with no prior policies. Also, the gap carries on after the SIPO expires. The figure suggests that more people shelter in highly populated areas. This may still be a reflection of the fact that the perceived risk of catching the disease is higher in those areas, and the impact of fear is strong enough to keep more people at home.

If the SIPO is effective in increasing the percentage of the population that stays at home, one would expect counties without any restrictive policies to catch up with others once the state-wide blanket policy is imposed. We estimate Equation (7) including all of the 254 counties. The results in Table 5 shows that the sheltering percentage is highest in the first week of the SIPO and gradually decreases, confirming our earlier results. However, there is no evidence that the sheltering percentage in the counties without any prior policy converges to the percentages in the counties with proactive policies. In contrast, there seems to be a gap between these groups of counties, which persists during the policy period.

**Table 5:** Sheltering percentages all Texas counties.

	**Sheltering %**	**Sheltering %**	**Sheltering %**
SIPO_0_	18.729^***^	19.075^***^	20.241^***^
	(0.679)	(0.672)	(0.644)
SIPO_1_	16.474^***^	16.798^***^	18.017^***^
	(0.687)	(0.673)	(0.635)
SIPO_2_	15.548^***^	15.946^***^	16.886^***^
	(0.628)	(0.612)	(0.634)
SIPO_3_	10.963^***^	11.341^***^	12.299^***^
	(0.613)	(0.577)	(0.550)
SIPO_0_*NoPolicy		−1.855^***^	−8.093^***^
		(0.563)	(1.066)
SIPO_1_*NoPolicy		−1.736^***^	−8.257^***^
		(0.542)	(1.084)
SIPO_2_*NoPolicy		−2.131^***^	−7.158^***^
		(0.472)	(1.010)
SIPO_3_*NoPolicy		−2.021^***^	−7.144^***^
		(0.424)	(0.929)
**Fixed effects**			
County	Yes	Yes	Yes
Day	Yes	Yes	Yes
Day-NoPolicy	No	No	Yes
Observations	29,718	29,718	29,718
Adjusted R^2^	0.934	0.936	0.945

***,**,* *Indicate significance at 1, 5, and 10%, respectively. Robust standard errors are reported in parentheses*.

## 4. Conclusion and Discussion

We analyze both state and county-level effects of the SIPO on the growth of cases and deaths. We find that growth and death rates are lower during the SIPO period. We also see that a significantly larger percentage of the population stays at home during this period. Thus, it is not surprising to see that the disease slows down in the period of the SIPO. The more interesting question is whether the SIPO caused stay at home percentages to increase. We find two pieces of evidence that goes against such causality. First, even though the highest sheltering corresponds to the first week of the SIPO, sheltering percentages steadily declined even though the policy was in effect (Figures 2, 3). This pattern might be very much a behavioral response to the emergence and the initial rapid spread of the disease. The fact that people start to see cases in their communities may create fear, and people respond by staying at home. However, as the duration of home stay gets longer, people might develop fatigue and start moving again. Second, we make use of the fact that some counties adopted local “safer at home” policies several days before the state-wide blanket policy. The sheltering percentages in these counties started to increase before the state-wide SIPO. However, we see a similar upward trend, albeit at a slower rate, in counties where there were no such policies. Also, we do not observe that these groups of counties converge to a similar sheltering percentage after the state-wide blanket policy. Instead, the counties without any policy (less populated counties with slower growth of cases) have a lower rate of sheltering than the other counties. There seems to be a gap in terms of sheltering percentages, and it persists during as well as after the policy period (Figure 4).

Our analyses show that the growth rate of COVID-19 cases and deaths decreases when a larger share of the population exercises social distancing by staying at home. However, we do not find evidence that the state-wide shelter-in-place order increased the percentage of the population that stays at home. The initial local conditions and county policies may have already encouraged people to stay at home. It may be a better strategy to reach out to the population and inform them about the current state of disease in their localities. On the other hand, policymakers also need to consider the fact that people may not be able to stay at home even if they want to if their employers ask them to get back to work in the absence of such policies. What we suggest is that imposing restrictive policies alone may not be enough to guarantee that people will exercise social distancing. Thus, such policies must take local conditions into account and be accompanied by the effort to inform and educate the public about the potential consequences of the disease and the situation in their communities.

Even though the current study has interesting policy implications, it has limitations. In particular, the results may not need to generalize to other states or countries. A similar evolution of the pandemic and similar policy rules may generate quite different behavioral responses from the public elsewhere. More local-level studies may be needed to check the generalizability of our conclusions.

## Data Availability Statement

Publicly available datasets were analyzed in this study. This data can be found here: https://covid19.unfolded.ai/, https://www.naco.org/covid19/topic/research-data.

## Author Contributions

All authors listed have made a substantial, direct and intellectual contribution to the work, and approved it for publication.

## Conflict of Interest

The authors declare that the research was conducted in the absence of any commercial or financial relationships that could be construed as a potential conflict of interest.

## Footnotes

^1^Centers for Disease Control and Prevention, (CDC) : https://www.cdc.gov/coronavirus/2019-ncov/faq.html#Spread.

^2^Social distancing, also called “physical distancing” is defined by the CDC as keeping a safe space (at least 6 feet) between yourself and other people who are not from your household.

^3^The data are consistent with the count of cases and fatalities from the CDC and John Hopkins University.

^4^Several studies rely on SafeGraph for social distancing measures. See (3, 14–17) among many others.

^5^The continuous-time version yields similar conclusions. We stick to the discrete version for expositional simplicity.

^6^According to the World Health Organization, the incubation period for COVID-19, which is the time between exposure to the virus (becoming infected) and symptom onset, is on average 5–6 days, however, can be up to 14 days: https://www.who.int/docs/default-source/coronaviruse/situation-reports/20200402-sitrep-73-covid-19.pdf?sfvrsn=5ae25bc7_6#:~:text=.

^7^Weather conditions also affect the sheltering percentages. More people are likely to stay at home during rainy or cold days than on sunny days. However, we are mainly interested in the effect of the SIPO on sheltering. As long as the weather is not correlated with the enactment of the policy, our estimates would still be unbiased (18).

^8^The fit of the regression (R^2^) is largest with 13–15 days lags, and we use 14 days.

^9^The Appendix shows we get similar results with county-level panel data.

^10^Harris county declared public health emergency on March 11, and stay at home order on March 24, which is 9 days prior to the state-wide policy. Similarly, Dallas county declared an emergency on March 12 and ordered residents to stay at home on March 23. Tarrant county's emergency declaration is on March 13, and the county-level SIPO date is March 24. Bexar county's declarations were on March 13 and March 23, respectively. For Travis county, the dates are March 17 and March 24, respectively.

## Appendix

Including all of the 254 Texas counties, we estimate the following regression:

(A1) $$\begin{array}{l} y_{i,t}=\beta_{0}+\alpha_{0}SIPO_{0}+\alpha_{1}SIPO_{1}+\alpha_{2}SIPO_{2}+\alpha_{3}SIPO_{3}+Time_{t}\\ \;+County_{i}+u_{i,t} \end{array}$$

where *y*_*i,t*_ is the growth rate of cases and fatalities in county *i* at time *t*. We control for time and county fixed effects, and we cluster standard errors at the county-level. All estimations are weighted by county population.

Time captures days after the first case rather than calendar days. This way, we are comparing counties at the same point in the evolution of the disease's spread. If the spread of the disease were strictly exponential, this choice would not matter since the growth rate of cases would be the same regardless of whether a county has a few cases or many cases. Since we know the strict exponentiality is not realistic, our choice provides better estimates. The results in Table A1 shows that the growth rate of cases is 0.053–0.079 points lower during the SIPO period. The growth rates of fatalities are 0.032–0.058 point lower.

**Table A1:** The growth of COVID 19 cases and fatalities.

	**Cases**	**Fatalities**
SIPO_0_	−0.053^***^	0.014
	(0.014)	(0.041)
SIPO_1_	−0.075^***^	−0.045^***^
	(0.013)	(0.015)
SIPO_2_	−0.079^***^	−0.058^***^
	(0.011)	(0.015)
SIPO_3_	−0.054^***^	−0.032^***^
	(0.007)	(0.010)
**Fixed effects**		
County	Yes	Yes
Time	Yes	Yes
Observations	19,343	8,231
Adjusted R^2^	0.353	0.151

***,** *Indicate significance at 1, 5, and 10%, respectively. Robust standard errors are reported in parentheses*.

Next, we look at the relation between the percentage of the population that stays at home in each county and the state-wide shelter-in-place order. We estimate Equation (A1) where *y*_*i, t*_ is the percentage of sheltering population. We control for county and time fixed-effects, cluster standard errors at the county level, and weight regressions by the counties' populations. The first column in Table A2 shows that the sheltering is 12.4 percentage points (almost 50%) higher in the first of the SIPO period. Even though the sheltering percentages are statistically larger in the following weeks as well, the margin decreases gradually. We also include dummies for 1 and 2 weeks before the SIPO to see how sheltering percentages look before the policy. The last two column reveals that the upward trend in sheltering percentages started even before the policy.

**Table A2:** Sheltering percentages and the SIPO.

	**Sheltering %**	**Sheltering %**	**Sheltering %**
SIPO_−2_			6.061^***^
			(0.879)
SIPO_−1_		12.542^***^	12.542^***^
		(0.597)	(0.597)
SIPO_0_	12.397^***^	12.397^***^	12.397^***^
	(0.635)	(0.635)	(0.635)
SIPO_1_	10.142^***^	10.142^***^	10.142^***^
	(0.649)	(0.649)	(0.649)
SIPO_2_	9.217^***^	9.217^***^	9.217^***^
	(0.614)	(0.614)	(0.614)
SIPO_3_	4.632^***^	4.632^***^	4.632^***^
	(0.591)	(0.591)	(0.591)
**Fixed effects**			
County	Yes	Yes	Yes
Time	Yes	Yes	Yes
Observations	29,972	29,972	29,972
Adjusted R^2^	0.933	0.933	0.933

***,** *Indicate significance at 1% and 5%, respectively. Robust standard errors are reported in parentheses*.

The SIPO is expected to slow down the spread of the disease by increasing social distancing in the population. So, we expect it to influence the spread of disease via its effect on the number of people sheltering in place. Due to the nature of the disease, a change in sheltering behavior impacts the spread of the disease with a lag. We estimate Equation (A1) controlling for the sheltering percentage 14 days prior. The independent variable *y*_*i,t*_ is the growth rate of cases or deaths. We include county and time dummies, and weigh regressions by county populations. Table A3 shows past sheltering percentages are statistically significant determinants of the current growth of cases and fatalities, while the SIPO dummies are no longer statistically significant.

**Table A3:** The Growth rates controlling for lagged sheltering percentages.

	**Cases**	**Fatalities**
SIPO_0_	−0.012	0.038
	(0.010)	(0.036)
SIPO_1_	−0.014	−0.014
	(0.012)	(0.014)
SIPO_2_	0.003	−0.019
	(0.011)	(0.014)
SIPO_3_	0.001	−0.006
	(0.008)	(0.009)
Lagged sheltering	−0.007^***^	−0.004^**^
	(0.001)	(0.001)
**Fixed effects**		
County	Yes	Yes
Time	Yes	Yes
Observations	19,286	8,231
Adjusted R^2^	0.403	0.155

***,** *Indicate significance at 1% and 5%, respectively. Robust standard errors are reported in parentheses*.

## References

[1] Saglietto A D'Ascenzo F Zoccai GB De Ferrari GM. COVID-19 in Europe: the Italian lesson. Lancet. (2020) 395:P1110–1. 10.1016/S0140-6736(20)30690-5 32220279 PMC7118630

[2] Yuan J Li M Lv G Lu ZK. Monitoring transmissibility and mortality of COVID-19 in Europe. Int J Infect Dis. (2020) 95:311–5. 10.1016/j.ijid.2020.03.050 32234343 PMC7102547

[3] Dave D Friedson AI Matsuzawa K Sabia JJ. When do shelter-in-place orders fight COVID-19 best? Policy heterogeneity across states and adoption time. Econ Inq. (2020). 10.1111/ecin.12944. [Epub ahead of print]. 32836519 PMC7436765

[4] Killgore WDS Cloonan SA Taylor EC Dailey NS. Loneliness: a signature mental health concern in the era of COVID-19. Psychiatry Res. (2020) 290:113117. 10.1016/j.psychres.2020.113117 32480121 PMC7255345

[5] Smith ML Steinman LE Casey EA. Combatting social isolation among older adults in a time of physical distancing: the COVID-19 social connectivity paradox. Front. Public Health. (2020) 8:403. 10.3389/fpubh.2020.00403 32850605 PMC7396644

[6] Bhutani S Cooper JA. COVID-19-related home confinement in adults: weight gain risks and opportunities. Obesity. (2020) 28:1576–7. 10.1002/oby.22904 32428295 PMC7276847

[7] Stoker S McDaniel D Crean T Maddox J Jawanda G Krentz N Effect of shelter-in-place orders and the COVID-19 pandemic on orthopaedic trauma at a community level II trauma center. J Orthop Trauma. (2020) 34:e336–42. 10.1097/BOT.0000000000001860 32815848 PMC7446991

[8] Bullinger LR Carr JB Packham A “COVID-19 and crime: effects of stay-at-home orders on domestic violence,” in NBER Working Paper 27667. Cambridge, MA (2020).

[9] Froimson JR Bryan DS Bryan AF Zakrison TL COVID-19, home confinement, and the fallacy of “safest at home”. Am J Public Health. (2020) 110:960–1. 10.2105/AJPH.2020.305725

[10] Kofman YB Garfin DR. Home is not always a Haven; The domestic violence crisis amid the COVID-19 pandemic. Psychol Trauma Theory Res Pract Policy. (2020) 12:S199–201. 10.1037/tra0000866 32478558 PMC7720288

[11] Sen S Karaca-Mandic P Georgiou A. Association of stay-at-home orders with COVID-19 hospitalizations in 4 states. JAMA. (2020) 323:2522Ű–4. 10.1001/jama.2020.9176 32459287 PMC7254451

[12] Wei L Wehby GL Shelter-in-Place orders reduced COVID-19 mortality and reduced the rate of growth in hospitalizations. Health Aff. (2020) 39:1615–23. 10.1377/hlthaff.2020.00719 32644825

[13] Courtemanche C Garuccio K Le A Pinkston J Yelowitz A. Strong social distancing measures in the United States reduced the COVID-19 growth rate. Health Affairs. (2020) 39:1237–46. 10.1377/hlthaff.2020.00608 32407171

[14] Friedson AI McNichols D Sabia JJ Dave D “Did California's shelter-in-place order work? Early Coronavirus-related public health effects,” in NBER Working paper 26992. Cambridge, MA (2020). Available online at: https://onlinelibrary.wiley.com/doi/abs/10.1111/ecin.12944

[15] Ashby NJS. Impact of the COVID-19 pandemic on unhealthy eating in populations with obesity. Obesity. (2020) 28:1802–5. 10.1002/oby.22940 32589788 PMC7361200

[16] Weill JA Stigler M Deschenes O Springborn MR. Social distancing responses to COVID-19 emergency declarations strongly differentiated by income. Proc Natl Acad Sci USA. (2020) 117:19658–60. 10.1073/pnas.2009412117 32727905 PMC7443940

[17] Woody S Garcia Tec M Dahan M Gaither K Lachmann M Fox S Projections for first-wave COVID-19 deaths across the US using social-distancing measures derived from mobile phones. medRxiv. (2020). 10.1101/2020.04.16.20068163

[18] Wooldridge JM Introductory Econometrics: A Modern Approach. 6th ed Cengage Learning (2016).

